# The endocannabinoid anandamide is a precursor for the signaling lipid *N*-arachidonoyl glycine by two distinct pathways

**DOI:** 10.1186/1471-2091-10-14

**Published:** 2009-05-21

**Authors:** Heather B Bradshaw, Neta Rimmerman, Sherry Shu-Jung Hu, Valery M Benton, Jordyn M Stuart, Kim Masuda, Benjamin F Cravatt, David K O'Dell, J Michael Walker

**Affiliations:** 1 The Department of Psychological and Brain Sciences, Indiana University, Bloomington, IN, USA; 2 The Gill Center for Biomolecular Science, Indiana University, Bloomington, IN, USA; 3 The Kinsey Institute for Research in Sex, Gender and Reproduction, Indiana University, Bloomington, IN, USA; 4 The Scripps Institute, La Jolla, CA, USA

## Abstract

**Background:**

*N*-arachidonoyl glycine (NAGly) is an endogenous signaling lipid with a wide variety of biological activity whose biosynthesis is poorly understood. Two primary biosynthetic pathways have been proposed. One suggests that NAGly is formed via an enzymatically regulated conjugation of arachidonic acid (AA) and glycine. The other suggests that NAGly is an oxidative metabolite of the endogenous cannabinoid, anandamide (AEA), through an alcohol dehydrogenase. Here using both *in vitro *and *in vivo *assays measuring metabolites with LC/MS/MS we test the hypothesis that both pathways are present in mammalian cells.

**Results:**

The metabolic products of deuterium-labeled AEA, D_4_AEA (deuterium on ethanolamine), indicated that NAGly is formed by the oxidation of the ethanolamine creating a D_2_NAGly product in both RAW 264.7 and C6 glioma cells. Significantly, D_4_AEA produced a D_0_NAGly product only in C6 glioma cells suggesting that the hydrolysis of AEA yielded AA that was used preferentially in a conjugation reaction. Addition of the fatty acid amide (FAAH) inhibitor URB 597 blocked the production of D_0_NAGly in these cells. Incubation with D_8_AA in C6 glioma cells likewise produced D_8_NAGly; however, with significantly less efficacy leading to the hypothesis that FAAH-initiated AEA-released AA conjugation with glycine predominates in these cells. Furthermore, the levels of AEA in the brain were significantly increased, whereas those of NAGly were significantly decreased after systemic injection of URB 597 in rats and in FAAH KO mice further supporting a role for FAAH in endogenous NAGly biosynthesis. Incubations of NAGly and recombinant FAAH demonstrated that NAGly is a significantly less efficacious substrate for FAAH with only ~50% hydrolysis at 30 minutes compared to 100% hydrolysis of AEA. Co-incubations of AEA and glycine with recombinant FAAH did not, however, produce NAGly.

**Conclusion:**

These data support the hypothesis that the signaling lipid NAGly is a metabolic product of AEA by both oxidative metabolism of the AEA ethanolamine moiety and through the conjugation of glycine to AA that is released during AEA hydrolysis by FAAH.

## Background

**Figure 1:**
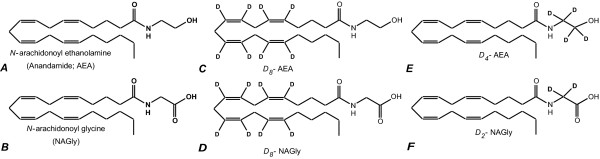
**Structures of AEA and NAGly**. A) the endocannabinoid, *N*-arachidonoyl ethanolamine (anandamide; AEA) and B) the related signaling lipid, *N*-arachidonoyl glycine (NAGly); C) deuterium-labeled AEA with eight deuteriums on the arachidonic acid moiety; D) deuterium-labeled NAGly with eight deuteriums on the arachidonic acid moiety; E) deuterium-labeled AEA with four deuteriums on the ethanolamine moiety; F) deuterium-labeled NAGly with 2 deuteriums on the glycine moiety.

*N*-arachidonoyl glycine (NAGly) was synthesized as part of a structure activity relationship study of the endocannabinoid anandamide (*N*-arachidonoyl ethanolamine; AEA; Fig. 1A) differing from AEA by the oxidation state of the carbon beta to the amido nitrogen (Fig. 1B); a modification that drastically reduces its activity at both cannabinoid receptors [1]. Nevertheless, NAGly produces antinociceptive and anti-inflammatory effects in mice and rats [2-5]. These findings gained physiological relevance when Huang et al. [3] demonstrated that NAGly is formed in numerous mammalian tissues including the brain. Subsequent studies by Kohno and colleagues [6] found that low concentrations (EC_50 _~20 nM) of NAGly activate GPR18, an orphan G protein-coupled receptor. Consistent with the anti-inflammatory effects of NAGly, GPR18 is highly expressed in peripheral blood leukocytes and several hematopoietic cell lines. In pancreatic beta cells, NAGly caused intracellular calcium mobilization and insulin release [7]. NAGly inhibited the glycine transporter, GLYT2a through direct, non-competitive interactions [8] and more recently was reported as a partial agonist of G_q/11_-coupled GPR92 receptors [9]. These data support the hypothesis that NAGly is an endogenous signaling molecule with multiple biological activities.

The biosynthesis and regulation of NAGly are only partially understood. Unlike 2-arachidonoyl glycerol and AEA, the biosynthesis of NAGly cannot logically be derived from phospholipid biochemistry. Two primary pathways for the biosynthesis of NAGly, have been proposed: 1) conjugation of arachidonic acid and glycine [2,3,10] and 2) oxygenation of AEA via the sequential enzymatic reaction of alcohol dehydrogenase (ADH) and aldehyde dehydrogenase [2,11].

Huang et al. [3] proposed that NAGly is synthesized by the condensation of arachidonic acid (AA) with glycine based upon the formation of deuterated NAGly following incubations of brain membranes with deuterated AA and deuterated glycine. McCue and colleagues [10] demonstrated that NAGly is formed via cytochrome C acting on arachidonoyl CoA and glycine in support of this conjugation pathway. Fatty acid amide hydrolase (FAAH), the primary hydrolyzing enzyme of AEA and other *N*-acyl amides [12], could potentially be involved in this reaction by acting in the biosynthetic pathway. In addition to being a hydrolytic enzyme, FAAH was suggested to play a role in the conjugation pathway of the biosynthesis of AEA from AA and ethanolamine [13] and was recently reported to participate in the synthesis of *N*-arachidonoyl 4-aminophenol (AM404) by conjugation of AA to exogenously administered *p*-acetamidophenol [14]. NAGly inhibited the hydrolysis of AEA by FAAH [3,15] indicating that it likely interacts with FAAH, presumably as a competitive substrate, though this interaction has not been fully examined.

An alternative pathway was proposed by Burstein et al. [2] who speculated that NAGly is produced by the oxidation of the ethanolamine in AEA, most likely through an ADH. Recent evidence using *in vitro *studies with human ADH confirmed this hypothesis by demonstrating that AEA is the precursor to NAGly through an aldehyde intermediate, *N*-arachidonoyl glycinal, which was synthesized and measured throughout the reaction [11]. These experiments show that after the reaction proceeds through to the carboxylic acid it cannot be then formed into the aldehyde supporting the hypothesis that AEA is a precursor for NAGly via ADH and further showing that the reaction cannot proceed in the opposite direction.

The potent actions of NAGly in a number of biological systems magnify the need to better understand its biosynthesis. Here using both *in vitro *and *in vivo *biochemical assays and measuring metabolites with LC/MS/MS we show that AEA serves as a precursor to NAGly by both oxidative metabolism of the ethanolamine moiety and through an additional conjugation pathway involving FAAH activity.

## Results

### Oxidative Metabolism of deuterium-labeled AEA in RAW 264.7 cells produces deuterium-labeled NAGly

**Figure 2:**
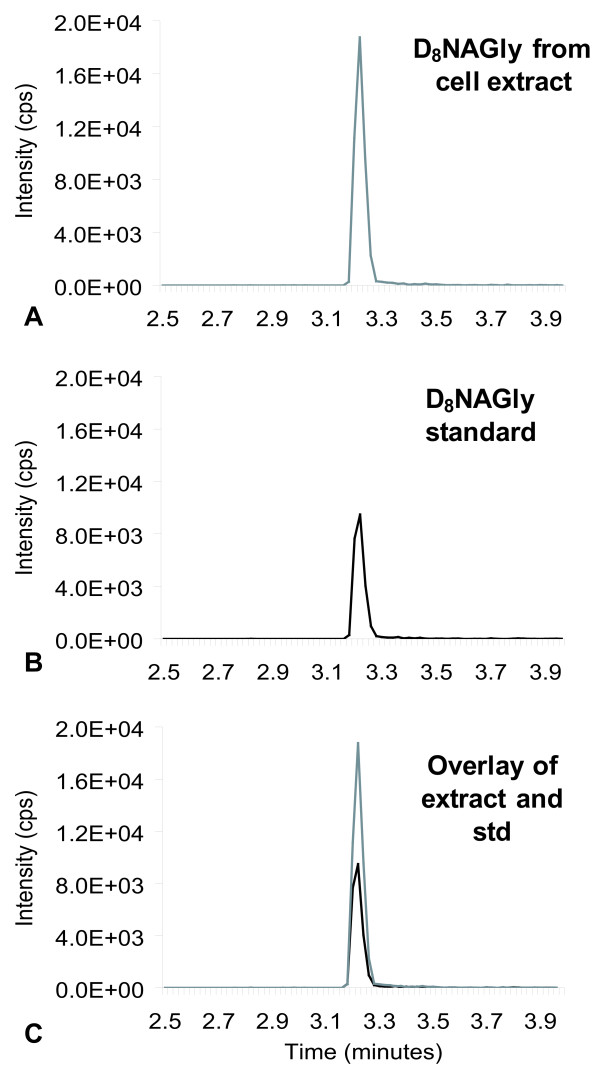
**Chromatograms of the tandem mass spectrometric (MS) method for deuterium-labeled NAGly (D_8_NAGly) in which the parent mass in negative mode [368.3]^- ^is paired fragment mass of glycine [74.0]^-^**. A) Chromatogram of an MS scan for the 368.3/74 pair from RAW 267.4 cell extracts that were incubated with deuterium-labeled anandamide. B) Chromatogram of a scan for the 368.3/74 pair with the synthesized D_8_NAGly standard (std). C) Overlay of the two independent scans.

Incubation of deuterium-labeled AEA, (D_8_AEA, deuterium labeled on the arachidonic acid chain; Fig 1C) with RAW 264.7 murine macrophage-like cells resulted in the production of deuterium-labeled NAGly (D_8_NAGly, deuterium labeled on the arachidonic acid chain; Fig 1D). This was demonstrated by the isolation and measurement of a product that has the exact retention-time and parent mass/fragment pairing as synthetic D_8_NAGly (368.3/74.2; Fig 2). Incubation of RAW 264.7 with D_8_NAGly did not result in the production of a compound with the chromatographic or mass spectrometric properties of D_8_AEA, nor did incubation of D_8_AA produce either D_8_AEA or D_8_NAGly *(data not shown)*. Because D_8_AA incubation alone did not produce D_8_NAGly, this suggested that conjugation of D_8_AA to glycine was not the biosynthetic pathway in this cell type. Furthermore, pre-incubation of RAW 264.7 cells with the FAAH inhibitor URB 597 for one hour followed by incubation with D_8_AEA did not block the production of D_8_NAGly. Therefore, we hypothesized that the conversion was on the ethanolamine moiety in AEA to form the glycine as was previously suggested [2,11].

**Figure 3:**
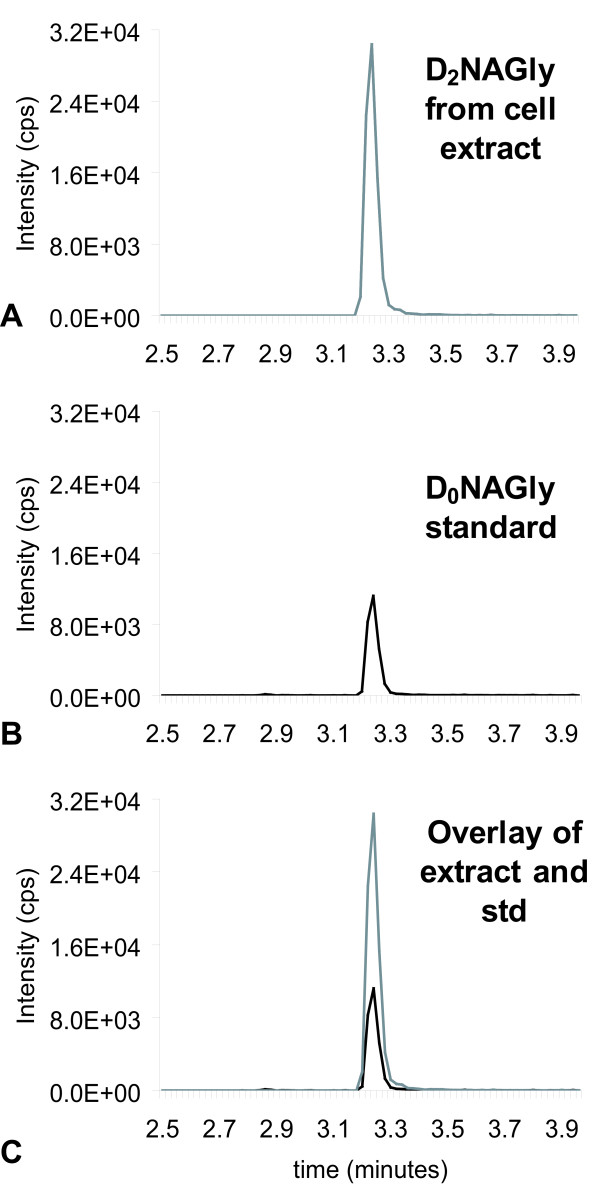
**Chromatograms of the tandem mass spectrometric (MS) method for deuterium-labeled *N*-arachidonoyl glycine (D_2_NAGly) in which the parent mass in negative mode [362.3]^- ^is paired fragment mass of deuterium-labeled glycine [76.0]^- ^or the non-deuterium-labeled *N*-arachidonoyl glycine (D_0_NAGly) that has a parent mass in negative ion mode of [360.3]^- ^and a paired fragment mass of glycine [74.0]^-^**. A) Chromatogram of an MS scan for the 362.3/76 pair from RAW 267.4 cell extracts that were incubated with deuterium-labeled anandamide (D_4_AEA). B) Chromatogram of the MS scan for the 360.3/74 pair with the synthesized D_0_NAGly standard (std). C) Overlay of the two independent scans.

**Figure 4:**
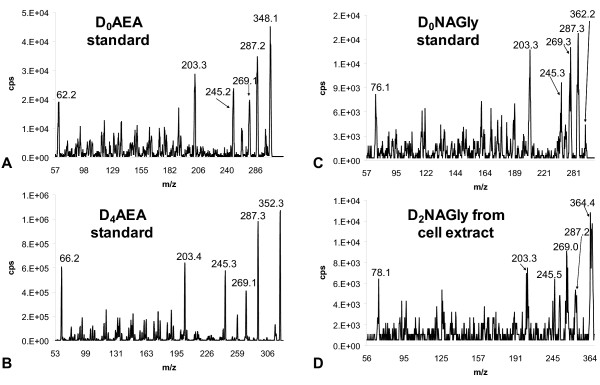
**Mass spectrometric product ion scans of synthesized standards and RAW 264.7 lipid extracts**. The numbers above the peaks are the calculated centroid mass. A) Positive ion mode product ion scan of the mass [348.3]^+ ^of the synthesized standard of non-deuterium labeled *N*-arachidonoyl ethanolamine (D_0_AEA). B) Positive ion mode product ion scans of the mass [352.3]^+ ^of the synthesized standard of deuterium-labeled AEA (D_4_AEA). C) Positive ion mode product ion scan of the mass [362.3]^+ ^of the synthesized standard of non-deuterium labeled NAGly (D_0_NAGly). D) Positive ion mode product ion scan of the mass [364.3]^+ ^of the RAW 264.7 cell extract that was incubated with D_4_AEA.

To test this hypothesis, the same series of incubations were performed using D_4_AEA (Fig. 1E), with the reasoning that the actions of an ADH on the D_4_ethanolamine moiety would yield a glycine with two deuterium atoms and would, therefore, produce D_2_NAGly (Fig. 1F). Incubation of D_4_AEA with RAW 264.7 cells yielded a product that matched the characteristics of the proposed D_2_NAGly (Figs. 3, 4). Chromatographic matches using HPLC/MS/MS showed that a molecule with the parent mass of the predicted D_2_NAGly (363.2 in negative ion mode) and a fragment that was 2 atomic mass units (amu) greater than the glycine fragment (76.1 and 74.1 respectively; Fig. 3A) had the identical retention time as the non-deuterated (D_0_NAGly) standard (Fig. 3B, C).

Product ion scans in positive ion mode (used to facilitate the generation of more fragments) established that the fragmentation pattern of non-deuterated AEA (D_0_AEA; Fig. 4A) and the deuterium-labeled AEA synthesized here, D_4_AEA (Fig. 4B), are identical with the exception of the parent mass increasing by 4 amu from [348.1]^+ ^to [352.3]^+ ^and ethanolamine fragment from [62.2]^+ ^and [66.2]^+ ^respectively. Likewise, positive ion scans of NAGly standard (Fig. 4C) are identical to the molecule produced after D_4_AEA incubation with the predicted addition of 2 amu on the parent mass [362.2]^+ ^and [364.4]^+ ^respectively and glycine fragments [76.1]^+ ^and [78.1]^+ ^respectively (Fig 4D). Major product ions (287, 269, 245, and 203 m/z), which are associated with the fragmentation of AA, were likewise the same in each of the four scans (Fig 4A–D). These findings provide further evidence that NAGly is produced via an ADH from AEA in RAW 264.7 cells by activity on the ethanolamine moiety rather than cleavage to AA which is subsequently conjugated with glycine.

### The Role of FAAH in the Biosynthesis of Deuterium-labeled-NAGly from Deuterium-labeled AEA in C6 glioma cells

**Figure 5:**
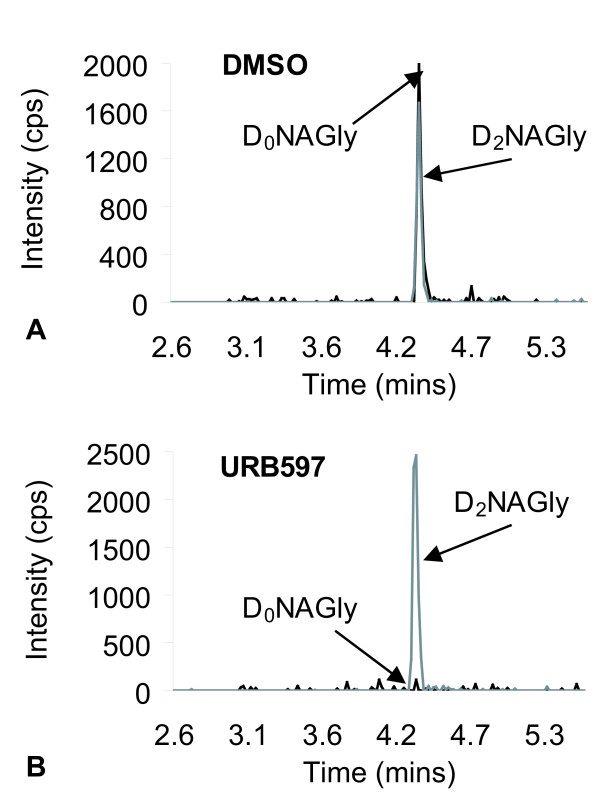
**Chromatograms of lipid extracts of C6 Glioma of the tandem mass spectrometric (MS) methods for both deuterium-labeled NAGly (D_2_NAGly) in which the parent mass in negative mode [362.3]^- ^is paired fragment mass of deuterium-labeled glycine [76.0]^- ^and the non-deuterium-labeled *N*-arachidonoyl glycine (D_0_NAGly) that has a parent mass in negative ion mode of [360.3]^- ^and a paired fragment mass of glycine [74.0]^-^**. A) Chromatogram of MS scans for D_2_NAGly and D_0_NAGly from the lipid extract of C6 Glioma cells that were incubated with DMSO followed by D_4_AEA. B) Chromatogram of MS scans for D_2_NAGly and D_0_NAGly from the lipid extract of C6 Glioma cells that were incubated with 1 μM URB 597 followed by D_4_AEA.

FAAH plays a role in the biosynthesis of *N*-arachidonyl-*p*-aminophenol following treatment of rats with acetaminophen [14]. Therefore, we hypothesized that FAAH may catalyze the biosynthesis of NAGly in C6 glioma cells, a murine cell line that exhibits robust FAAH activity [16,17]. D_8_AEA and D_4_AEA were incubated with C6 glioma cells using the treatment protocols described above for RAW 264.7 cells. Compounds matching the retention times and mass spectrometric properties of both D_8_NAGly and D_2_NAGly were present in the respective cell extracts. Unlike the RAW 264.7 cells, C6 glioma cells also produced excess D_0_NAGly after incubation with D_4_AEA (Fig 5A). The production of D_0_NAGly was prevented by pre-incubation with the FAAH inhibitor, URB 597 (Fig. 5B), however, like with the RAW 264.7 cells, D_2_NAGly was still produced (Fig 5B).

**Figure 6:**
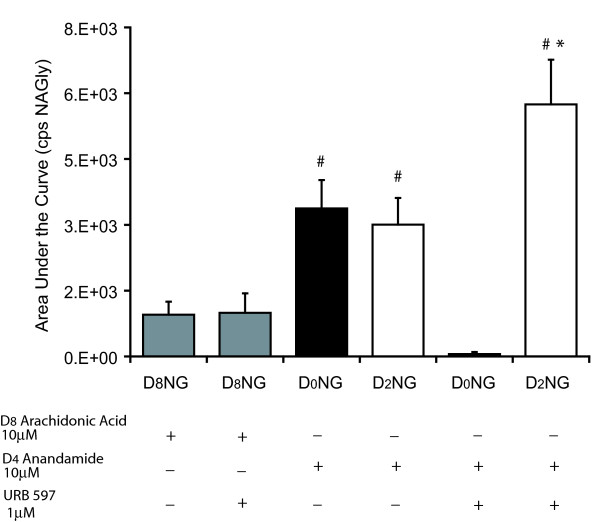
**Comparison of NAGly production in C6 glioma cells after incubation with deuterium-labeled arachidonic acid (D_8_AA) or ethanolamine moiety deuterium-labeled AEA (D_4_AEA)**. The products measured were arachidonoyl chain deuterium-labeled NAGly (D_8_NG); non-deuterium-labeled NAGly (D_0_NG); and glycine moiety deuterium-labeled NAGly (D_2_NG). + denotes an addition of the compound to the cell media for 1 hour before lipid extraction. -denotes compounds that were not present during the incubation. # p ≤ 0.05 compared to levels of D_8_NG in D_8_AA treatment group; * p ≤ 0.05 compared to levels of D_2_NG in D_4_AEA+ URB 597 treatment group; n = 6–8 per group.

In contrast to RAW 264.7 cells, D_8_AA incubated with C6 glioma cells yielded D_8_NAGly, however, this product was not blocked by the addition of URB 597 (Fig. 6). Furthermore, a comparison of D_8_AA versus D_4_AEA indicated that mole-for-mole AEA is a significantly better substrate for the biosynthesis of NAGly in C6 glioma cells than AA (Fig 6). Significantly, blocking FAAH-dependent production of NAGly with URB 597 produced an increase in the amount of D_2_NAGly in these cells (Fig 6). Indeed, the shunting of substrate to the ADH pathway was very efficient, evidenced by the observation that the total production of NAGly was the same with and without URB 597 (Fig. 6).

### Brain levels of AEA and NAGly after URB 597 injections in rats and in FAAH knockout (KO) and wild-type (WT) mice

**Figure 7:**
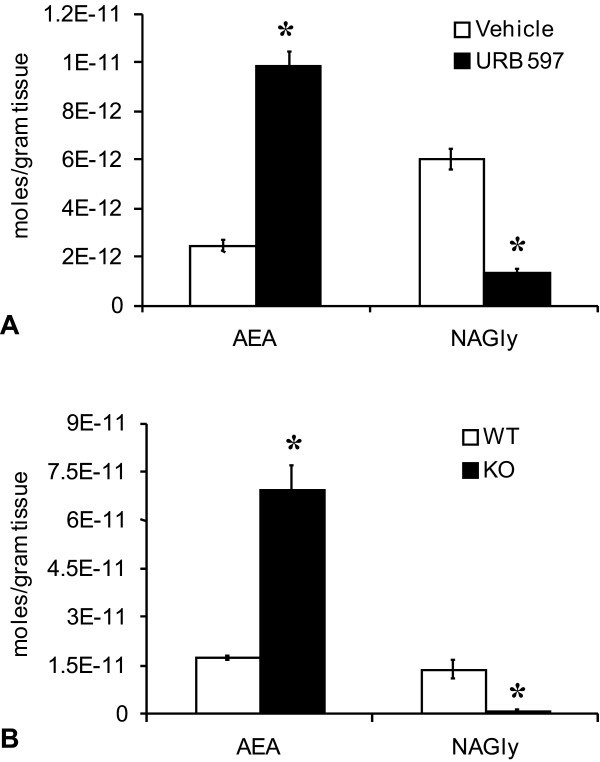
**Production of *N*-arachidonoyl ethanolamine (AEA) and *N*-arachidonoyl glycine (NAGly) in whole brain**. A) Levels of AEA and NAGly in rat whole brain two hours after vehicle (white bar) or 3 μg/kg URB 597 (black bar). * p ≤ 0.05 B) Levels of AEA and NAGly in mouse whole brain of WT (white bar) or FAAH KO (black bar). * p ≤ 0.05.

After examining the biosynthesis of NAGly in a FAAH-rich *in-vitro *cellular model (C6 glioma), we sought to determine whether FAAH-dependent biosynthesis of NAGly occurs also *in vivo*. We examined the levels of AEA and NAGly in brains of rats treated with URB597 or vehicle as well as in FAAH KO and WT mice. The levels of AEA in rat whole brain significantly increased 2 hours after systemic injection of URB 597 (0.3 mg/kg) compared to vehicle controls (Fig 7A). In contrast, levels of NAGly significantly decreased (Fig 7A). The same pattern was shown in the levels of AEA and NAGly in FAAH KO and WT mice: AEA levels were significantly higher in FAAH KO mice, whereas, NAGly levels were significantly lower (Fig. 7B).

### NAGly hydrolysis by recombinant FAAH

**Figure 8:**
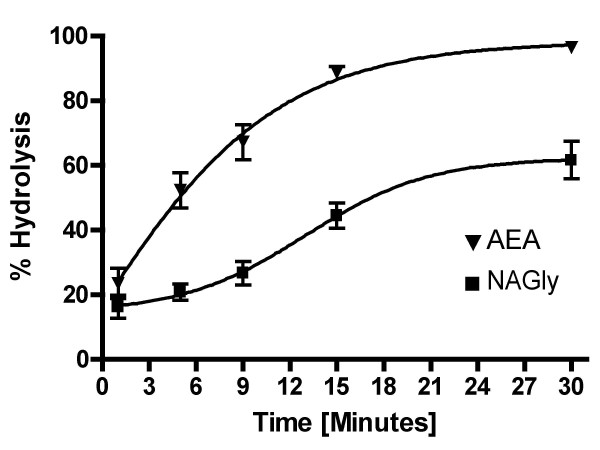
**Comparison of hydrolysis rates of *N*-arachidonoyl ethanolamine (AEA) and *N-*arachidonoyl glycine (NAGly) via recombinant FAAH**. n = 4–6 per time point. Time points (minutes): 1, 3, 5, 9, 15, 30.

The evidence that NAGly levels were significantly decreased with FAAH inhibition and in FAAH KO mice led us to test the hypothesis that: 1) FAAH is acting as a biosynthetic enzyme with AEA and glycine as precursors and 2) FAAH has a low efficacy for NAGly. If FAAH is highly efficacious for producing robust levels of NAGly hydrolysis, then it is unlikely to play a role in its biosynthesis. Conversely, if AEA is converted to NAGly during hydrolysis in the presence of glycine, then FAAH would be a candidate enzyme for NAGly biosynthesis. Our results show that when recombinant FAAH was incubated with AEA and glycine, AEA was measured via HPLC/MS/MS and was shown to be rapidly hydrolyzed as expected (Fig 8). At 5 minutes ~60% of all AEA was hydrolyzed and by 15 minutes over 95%. At 30 minutes there was almost no detectable AEA present in the solution. Conversely, at 5 minutes less than 20% of NAGly was hydrolyzed and at 30 minutes there was still over 45% of NAGly still left in the solution. There was no detectable NAGly produced in incubations of AEA, glycine and recombinant FAAH indicating that FAAH is not a direct biosynthetic enzyme in the recombinant form.

## Discussion

**Figure 9:**
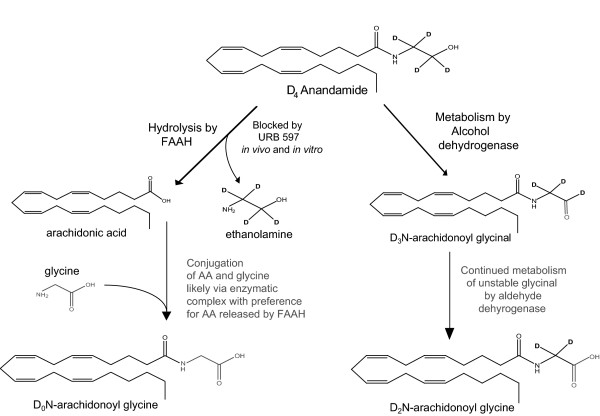
**Two pathways for *N*-arachidonoyl glycine (NAGly) biosynthesis from *N*-arachidonoyl ethanolamine (AEA)**.

The data presented here supports the hypothesis that the endogenous cannabinoid AEA acts as a precursor in the biosynthesis pathways of the signaling lipid NAGly. One pathway is a FAAH-dependent conjugation of glycine to AEA-released AA and the second is by oxidation of the ethanolamine moiety in AEA, likely by an ADH (Fig. 9).

The conjugation of AA with glycine to form NAGly demonstrated by Huang and colleagues [3] was confirmed here in the incubation with C6 glioma cells. The previous assay was performed with brain membranes and is more comparable to the brain-derived C6 glioma cells than the macrophage RAW 264.7 cell line. As shown here, AEA had a 4-fold greater efficacy as a substrate than AA in C6 glioma cells and the production of NAGly by conjugation of AA and glycine was not blocked by URB 597. Therefore, the present data suggest that AEA is a substrate for NAGly biosynthesis through the URB 597-sensitive pathway in C6 glioma cells and brain. Through this pathway, AEA must undergo hydrolysis by FAAH to be a substrate for NAGly biosynthesis via conjugation.

Previously, we demonstrated that incubation of D_8_AEA in a neuronal cell line (F-11) resulted in accumulation of D_8_AEA in lipid rafts while its metabolite D_8_AA was found mostly in non-lipid raft fractions [18]. It is possible that the trafficking of AEA from specialized membrane compartments such as lipid rafts to compartments rich in FAAH [19] may position the AA precursor in proximity to additional enzymes that are involved in the conjugation reaction to glycine. In essence, this would make the arachidonic acid in AEA more bio-available to form NAGly. Recently, McHue and colleagues [10] proposed that cytochrome c catalyzes the synthesis of NAGly from arachidonoyl CoA and glycine in the presence of hydrogen peroxide suggesting yet another conjugation pathway for the production of NAGly. These enzymes and FAAH are present in mitochondrial membranes, which may be the site of NAGly biosynthesis.

The conversion of AEA to NAGly through an ADH pathway in both RAW 264.7 macrophage and C6 glioma cell lines suggests a more ubiquitous biosynthesis reaction. Given that there are multiple members of the protein family of ADHs that act with different affinity to different substrates [20] it would also not be surprising that the level of NAGly production would be different with different cell types through this pathway.

We observed that incubation of D_8_NAGly with RAW 264.7 and C6 glioma cells did not lead to the production D_8_AEA. This finding is at variance with the report by Burstein et al. [21] in which incubation of NAGly with RAW 264.7 cells yielded increased levels of AEA. In the present study we used 5 cm^2 ^flasks for each assay which was an amount of cells that fell below the limit to detect endogenous AEA. In addition, the concentration of AEA in the incubations was 10-times less than in the earlier study. In light of the data presented here Burstein et al.'s result [21] may represent a case where NAGly at higher concentrations may be activating a pathway for AEA production or sufficiently blocking its enzymatic hydrolysis to cause a build-up of native AEA and not acting as a substrate for AEA synthesis.

That the levels of NAGly were dramatically decreased in brain after URB 597 and in the FAAH KO mice suggests that the compensatory NAGly production shown in the C6 glioma cells may be an acute phenomenon specific for these cells. If NAGly production were driven by ADH in the brain then the excess AEA generated by URB 597 should have produced an increase in NAGly. The lack of increase in this experiment and the FAAH KO mice suggests that the brain-derived NAGly is primarily through the FAAH-dependent conjugation biosynthesis pathway.

Finally, the evidence that recombinant FAAH has a significantly lower efficacy for NAGly hydrolysis than AEA suggests that it would not be readily hydrolyzed in an enzyme complex including FAAH permitting time for production and trafficking of the signaling molecule to its site of action, which is likely to be at plasma membrane receptors.

## Conclusion

Growing evidence supports a mechanism for non-CB_1_, non-CB_2 _activity of AEA [22-24]. The hypotheses generated from those studies were that AEA is acting on a separate receptor or receptors or through metabolites of AEA [24]. Here, we provide evidence that AEA is metabolized into the signaling lipid NAGly that activates GPR18 [6] and GPR92 [9] suggesting the hypothesis that non-CB receptor effects of AEA are potentially through this bioactive metabolite.

## Methods

### Subjects

Twelve male (300–450 g) Sprague-Dawley (Harlan, Indianapolis, IN) rats were used. We also used brain tissue from six FAAH WT and six FAAH KO mice, which were littermates from the thirteenth generation offspring from intercrosses of 129SvJ-C57BL/6 FAAH (±) mice [25]. All protocols were approved by the Indiana University Institutional Animal Care and Use Committee.

### Cell Culture

The RAW 267.4 and C6 glioma cell lines were purchased from ATCC (Manassas, VA). Both cell lines were cultured in DMEM (Mediatech, VA) with 10% fetal bovine serum (Hyclone, Logan, UT) and 1% penicillin-streptomycin (Gibco-Invitrogen, Carlsbad, CA).

### Drugs and reagents

HPLC-grade methanol and acetonitrile used for mass spectrometric studies were purchased from VWR international (Plainview, NY). HPLC grade water, mass spectrometry/HPLC grade acetic acid, formic acid, and ammonium acetate were purchased from Sigma-Aldrich (St. Louis, MO). *N*- [^2^H_8_]arachidonoyl glycine (D_8_NAGly), *N*- [^2^H_8_]arachidonoyl ethanolamine, D_8_AEA, and [^2^H_8_] arachidonic acid (D_8_AA) were purchased from Cayman Chemical (Ann Arbor, MI). URB 597 was purchased from BIOMOL International (Plymouth Meeting, PA). Arachidonic acid was purchased from Nu-Chek Prep (Elysian, MN). Ethanol-1,1,2,2-d4-amine was purchased from CDN Isotopes Inc. (Pointe-Claire, Quebec).

### Synthesis of deuterium-labeled N-arachidonoyl ethanolamine (D4-AEA)

D_4_AEA was synthesized as previously described with the exception that the ethanolamine was deuterium labeled instead of the arachidonic acid [26].

### Characterization of metabolites of D8AEA and D4AEA in cell culture systems

RAW 264.7 and C6 glioma cell lines were used at 70% confluence. Cells were washed twice with dPBS then either 1 μM of either D_8_AEA, D_4_AEA, D_8_AA, D_8_NAGly or vehicle, DMSO (10 μl) were added to serum-free media and incubated for 1 hour. In experiments in which the FAAH inhibitor URB 597 was used, the cells were first incubated with 1 μM URB 597 or DMSO vehicle (10 μl) in serum-free media for one hour prior to D_8_AEA or D_4_AEA that was added directly to this media. Then, equal volumes of methanol were added to the flasks; cells were scraped, aspirated, and centrifuged at 2000 × g for 15 min at 24°C. Supernatants were collected and HPLC grade water was added to make a 30% organic solution. Lipids were partially purified on C18 solid phase extraction columns as previously described [27]. In brief, each 500 mg column was conditioned with 5 ml methanol and 2.5 ml water followed by loading of the water/supernatant solution. Columns were then washed with 2 ml water and 1.5 ml 55% methanol. Compounds were eluted with 1.5 ml methanol. Eluants were vortexed at maximum speed prior to mass spectrometric analysis.

Rapid separation of analytes was obtained using 10 μl injections (Agilent 1100 series autosampler, Wilmington, DE) onto a Zorbax eclipse XDB 2.1 × 50 mm reversed phase column. Gradient elution (200 μl/min) was formed under pressure on a pair of Shimadzu (Columbia, Maryland) 10AdVP pumps. Mass spectrometric analysis was performed with an Applied Biosystems/MDS Sciex (Foster City, CA) API 3000 triple quadrupole mass spectrometer equipped with an electrospray ionization source. Levels of each compound were analyzed by multiple reactions monitoring (MRM) on the LC/MS/MS system. Mass spectrometric conditions were optimized for each compound using direct flow injection of synthetic standards of each compound. Product ion scans were performed with identical chromatographic conditions as the MRM scans with identical ionization and collision energy.

Eluants were tested for levels of D_8_AEA, D_4_AEA, D_8_NAGly, D_2_NAGly, (Fig. 1) and D_8_AA using MRM tandem mass spectrometric methods with parent and fragment ions as follows: in positive ion mode, AEA 348.3 → 62.3, D_8_AEA 356.3 → 62.3 and D_4_AEA 352.3 → 66.3; in negative ion mode, NAGly 360.2 → 76.2, D_8_NAGly 368.2 → 76.2, D_2_NAGly 362.2 → 78.2, and D_8_AA 311.5 → 267.3. Product ion scans were performed for D_0_NAGly and D_2_NAGly using the API 3000 scanning in positive ion mode for the products of 362.2 and 364.2 respectively.

### Quantification of tissue levels of AEA and NAGly

Each of the analytes was extracted and quantified using methods reported [27]. In brief, whole brains were dissected and flash-frozen in liquid nitrogen prior to lipid extraction, at which time twenty volumes of ice-cold methanol and 100 pmol D_8_NAGly (internal standard) were added to the methanol-tissue sample. The samples were maintained on ice and homogenized via polytron for 2 min and centrifuged for 20 min at 40,000 × g at 24°C. Supernatants were transferred to polypropylene 50 ml centrifuge tubes (VWR, Plainview, NY) and HPLC grade water was added to each sample to create a 70:30 (water:supernatant) mixture. Partial purification on C18 solid phase extraction and mass spectrometric analyses were identical to that described above.

### Effects of inhibition of FAAH on brain levels of AEA and NAGly

Animals were injected with either URB 597 (0.3 mg/kg, in 1% DMSO, i.p.) or vehicle. After two hours animals were decapitated and brains were dissected and flash-frozen in liquid nitrogen, extracted, purified and analyzed as described above.

### Analysis of brain extracts from FAAH KO and WT mice

FAAH KO and WT mice were sacrificed when they were 6 weeks old. The brains were dissected and stored at -80°C until used. Lipid extraction, partial purification, and quantitation were performed using the methods described above.

### Recombinant FAAH cell-free assay

To determine the rates of AEA and NAGly hydrolysis a solution of ethanol and compound (400 μM, 10 μl) was added to a solution of recombinant FAAH protein (10 μl, 1.3 μg/μl in 20 mM HEPES (pH 7.8), 150 mM NaCl, 10% glycerol, 1% Triton X-100) in buffer (Tris/EDTA, 380 μl, pH 9) at room temperature. A 40 μl aliquot of the reaction mixture was taken at appropriate time points and quenched with 1 ml of MeOH. To control for loss of AEA and NAGly to the sides of the tube and into micelles in the aqueous buffer, equal numbers of controls were run at the same time without FAAH. 1 μl of the quenched solution from each (FAAH incubations and controls) was analyzed by LC/MS/MS mass spectrometry as discussed above. Hydrolysis rates were determined by the average values of the analyte measured from the FAAH incubations subtracted from the average values of the controls (incubations with buffer and no FAAH) at each time point.

### Data Analysis

#### Mass spectrometric quantitation

The quantitation of analytes was achieved using Analyst software (Applied Biosystems-MDS Sciex; Framingham MA), which quantifies the amount of analyte in the sample based upon a power fit of a linear regression of known concentrations of synthetic standards. Those data were then analyzed as vehicle verses drug in the case of the URB 597 and as FAAH KO verses WT. Statistical differences were determined using ANOVA with post-hoc Fisher's LSD using a 95% confidence interval for the mean (SPSS software, Chicago, IL). Data are presented as mean ± standard error of the mean where *p *≤ 0.05 was considered statistically significant

## List of abbreviations

(D_8_NAGly): *N*- [^2^H_8_]arachidonoyl glycine; (D_8_AEA): *N*- [^2^H_8_]arachidonoyl ethanolamine; (D_8_AA): [^2^H_8_] arachidonic acid; (D_2_NAGly): *N*- [^2^H_2_]arachidonoyl glycine; (D_4_AEA): *N*- [^2^H_4_]arachidonoyl ethanolamine; (AEA): arachidonoyl ethanolamide; (NAGly): *N*-arachidonoyl glycine; (AA): arachidonic acid; (FAAH): fatty acid amide hydrolase.

## Authors' contributions

HB conceived of the project, developed culture and MS method, analyzed data and wrote the manuscript, NR performed C6 glioma culture experiments and edited the manuscript, S S-J H performed *in vivo *URB 597 and FAAH KO/WT MS experiments, VB and JS performed recombinant FAAH MS experiments, KM genotyped the FAAH KO and WT mice, BC created and maintained the FAAH KO mice in his laboratory, DO synthesized deuterium-labeled anandamide, JMW took part in planning and project development. This work was supported by DA01822, J Michael Walker.

## Acknowledgements

We dedicate this manuscript to J Michael Walker (1950–2008) whose love of science and life was an inspiration to us all.
